# ‘This has given me the peace of mind I needed’: ethnographic insights into Barrett’s oesophagus screening using the capsule sponge test

**DOI:** 10.1136/jme-2024-109928

**Published:** 2024-07-02

**Authors:** Elspeth Davies

**Affiliations:** 1 Social Anthropology, University of Cambridge, Cambridge, UK

**Keywords:** Ethics, Quality of Health Care

## Abstract

In 2021 and 2022, researchers carried out an implementation trial that considered how the capsule sponge test might be used to screen for Barrett’s oesophagus using a mobile clinic in East Anglia. This paper offers insights from 15 months of ethnographic fieldwork studying the trial. It aims to highlight the value of the test in offering reassurance to worried patients, particularly to those with a family history of oesophageal adenocarcinoma. It also considers the variety of aims people held for the capsule sponge test, including the hope that it would address their symptoms of acid reflux, and the conflict that sometimes emerged as a result. The second half of the paper uses fieldwork carried out in virtual support groups for people with Barrett’s oesophagus to explore experiences postdiagnosis, which sometimes were defined by fear of future cancers. It describes notable differences between the care offered to people with morphological risk conditions like Barrett’s oesophagus and the care given to those with genetic risk conditions, including the provision of genetic counselling. More broadly, the paper highlights a tension between patient-centred and risk-centred medicine that is likely to grow as healthcare services continue to shift towards preventative approaches.

## Introduction

 Barrett’s oesophagus (BO) is a metaplastic change in the lining of the oesophagus in which normal squamous epithelium is replaced by specialised or intestinalised columnar epithelium.^1^ Triggered by chronic exposure of the oesophageal lining to gastro-oesophageal reflux, BO is a known risk factor or precancer for oesophageal adenocarcinoma (OAC). Overall survival rates for this type of cancer are poor, with 12% of people surviving for more than 10 years after diagnosis.^2^ This is usually attributed to the late stage at which many oesophageal cancers are diagnosed—one audit stated that 44.9% of diagnoses in 2020/2021 were stage 4.^3^ A diagnosis of BO is thought to offer an opportunity to improve survival rates. Those diagnosed with the condition in the UK are offered regular white light endoscopic surveillance, usually every 2–5 years depending on the severity of their BO.^4^ Surveillance aims to detect dysplasia, which can be treated with endoscopic therapy to prevent the development of OAC.

At present, the diagnosis of BO relies on the use of endoscopy, which is invasive and expensive. Researchers in recent decades have sought to develop easier ways to diagnose the condition without endoscopy, such as using a capsule sponge or ballon device linked with biomarker tests. The capsule sponge is a pill on a string that patients swallow. The coating of the capsule dissolves in the stomach to reveal a sponge, which collects cells from the lining of the oesophagus when it is retrieved. These cells then undergo laboratory analysis for biomarkers that indicate the presence of intestinal metaplasia and progression towards cancer demarcated by abnormalities of the p53 gene and dysplasia, with alternative molecular tests including methylation undergoing evaluation. The capsule sponge has a substantial evidence base and is now endorsed by some society guidelines for the diagnosis of BO,^5 6^ with trials ongoing to evaluate its use for proactive screening and BO monitoring.^7^,^10^ In 2021 and 2022, researchers used a mobile clinic with the aim of offering the test to 1500 people in three demographically distinct locations in the East of England. In this trial, the capsule sponge test was offered to people with a history of acid reflux in particular age brackets who were deemed to be at an increased risk of BO and OAC.

Diagnosed and monitored due to its potential for future harm rather than current symptoms, such efforts to screen for BO exemplify a broader shift towards risk-centred medical practice that has occurred in recent decades in some parts of the world, including in the UK.^11^,^13^ In the past, people became patients only when they felt unwell and visited their doctor seeking remedies to help them feel better. In the 1970s, however, clinicians developed a new aim—not only did they seek to alleviate suffering in the present, but they also aspired to reduce people’s risk of developing diseases in the future (ibid). This shift involved intervening in seemingly healthy people before they became sick. A new notion of care has arisen as a result—one that would have seemed unintelligible to people a century ago. Social scientists and historians have written extensively about this shift towards risk-centred medicine (eg,^1 2^), but there remains a need for ethnographic research highlighting its day-to-day consequences. This paper aims to do so by studying clinician scientists working to diagnose and patients learning to live with BO.

## Methods

### Design and data collection

The author carried out ethnographic fieldwork in capsule sponge test clinics that were conducted as part of the implementation trial. She attended clinics 1 day a week between September 2021 and September 2022 where she observed clinical interactions and took anonymised fieldnotes. Permission to observe appointments for research purposes was gained using a written consent form.

All patients were asked if they would like to participate in an interview. The author conducted interviews with 50 patients, including roughly equal numbers of people in each location. Semistructured interviews were carried out after patients had received their results, usually 2–3 weeks after their appointment. Interviews were repeated several times over a period of 6–12 months with 10 patients.

Participant observation was also carried out in several Facebook support groups for people with BO between June 2021 and December 2022. 25 members of these groups were interviewed. Most interviews took place over videocall, with a minority conducted over the telephone or in person. Participants provided written consent for participation. Interviews were recorded before being transcribed and the recording was deleted.

The author interviewed five researchers involved in the project, as well as conducting ethnographic observations at many public events about the capsule sponge test, and about cancer early detection technologies more generally, in an effort to grasp biomedical perspectives on the device. Research papers and guidelines were similarly studied with this aim.

### Qualitative analysis

Fieldnotes and interview transcripts were analysed and coded thematically with reference to a range of anthropological theoretical frameworks that seek to examine the social context in which people become patients, receiving and living with diagnoses. In particular, the paper draws on the work of scholars who have understood diagnosis as a social and material process^14^,^16^ and embodiment theorists who have described the lived and felt experiences of health and disease.^17^ All data collection and analysis was carried out by the author, who is a non-clinically trained ethnographer and who was a PhD student in Social Anthropology throughout the project.

## Results and discussion

Insights have been divided into two categories: the first relates to people’s hopes for the capsule sponge test, and the second to the care provided to patients once they are diagnosed with BO. The third section offers suggestions for improving future care.

### The aims of screening

‘After what happened to my mum, it was really reassuring to receive a negative result and has put my mind at rest,’ Sarah explained to me. She had recently participated in the implementation trial for the capsule sponge test. A decade ago, her mother died 6 months after being diagnosed with stage 4 OAC, leaving Sarah worried about her own risk of facing the disease in the future. She was pleased that the new device could offer a method for examining the oesophagus that was quicker and easier than an endoscopy and so to participate. After undergoing the capsule sponge test procedure, Sarah found the period of waiting for results anxiety-inducing and described feeling nervous each morning until the post arrived.^18 19^ Two weeks later, however, a letter was delivered stating that nothing abnormal had been found by the test. Sarah described her relief: ‘this has given me the peace of mind I needed.’

Research into patient experiences of other types of cancer screening has similarly highlighted their valuable role in offering reassurance.^20 21^ In comparison, however, awareness surrounding oesophageal cancer is relatively low,^22^ meaning that people were often less concerned about it than other types of cancer. For those who did not have personal experiences of losing loved ones to OAC, receiving a negative result after undergoing the capsule sponge test could offer reassurance, but seemingly somewhat only because worry had been provoked by the invitation to be screened.^11^ The possible self-fulfilling nature of reassurance in this case may require further research.

Furthermore, although important to many patients, reassurance was not the primary aim of the capsule sponge test according to the researchers involved. This tension materialised when patient and public involvement (PPI) representatives wanted to expand the inclusion criteria to offer the test to younger people who were statistically at lower risk of oesophageal cancer, but who were worried about their cancer risk. In contrast, researchers felt that the test needed to be aimed at those most at risk. One explained: ‘these younger people won’t benefit. They’re just looking for reassurance’. But to PPI representatives, reassurance was a benefit. Biomedical professionals also asserted the need to exclude low-risk groups in order to minimise harms such as false positives. Patients who received false positive results, however, often felt they had benefitted from the interventions, having found it reassuring to be tested. This may raise questions about what constitutes the ‘harms’ and ‘benefits’ of screening and who gets to decide.

Those who knew little about oesophageal cancer and its poor survival statistics experienced the screening test differently from people like Sarah. Prior to being invited to participate in the trial by his general practitioner (GP), John had never heard of this cancer type. He had been taking omeprazole for 18 years in order to manage his persistent symptoms of heartburn. In recent years, John’s GP had increased his dosage because the medication had become less effective at alleviating the burning sensation he felt in his chest at night. In contrast to the sense of relief experienced by Sarah, John was frustrated when he received a negative result several weeks after undergoing the procedure. He explained his disappointment at being discharged from the capsule sponge project: ‘they said I was all clear, but I wasn’t all clear. I was in pain.’ Risk management was not important to John in the same way as it was for Sarah, who had become acutely concerned about her risk of this disease after her mother’s death. What mattered most to John was his embodied experience of suffering in the present—which he felt was somewhat overlooked by the screening test.

To the researchers working on the capsule sponge test, the aim was to use it to decide whether monitoring or endoscopic treatment was needed in order to prevent cancer. Acid reflux symptoms should be dealt with by patients’ GPs. Nevertheless, psychosocial studies concerning other screening tests have highlighted that some patients understand screening as intended for people who need help with symptoms^23^—as we saw in John’s case. This is particularly an issue in BO screening because, unlike in other cancer types, symptoms function as a risk factor for disease so people being screened are symptomatic. Measures to help patients manage their symptoms of acid reflux were important to these patients; many were grateful for the leaflet on symptom management that was offered at the end of their appointments, which helped most feel like these symptoms were being taken seriously. Such measures may help screening interventions feel more ‘patient-centred’.

### The contested aims of postdiagnosis care

Care for people with BO primarily consists of endoscopic surveillance, ethnographic fieldwork highlighted that patients’ embodied experiences in the present can be overlooked in the process, including their anxiety about future cancers. For example, when I first met Janet her diagnosis of BO the previous year had left her acutely worried about the possibility of facing future OAC. Janet was most concerned that she would not live long enough to see her children grow up; the strong association between cancer and death has a long history in the UK^24^ and persists to this day. She felt as if cancer, and therefore, also death, was an impending inevitability. Even a year after her diagnosis, she was still experiencing significant anxiety surrounding her BO. She spent hours each week researching the diagnosis and was struggling to sleep.

Janet’s experience is by no means universal, although psychosocial studies have highlighted that it is not uncommon for diagnoses of BO to cause significant anxiety.^25^ Researchers have argued that these diagnoses can lead to a decrease in health-related quality of life (HRQL), which is usually attributed to the burden of living with a premalignant condition and the experience of suffering with symptoms of gastro-oesophageal reflux disease (GORD), a risk factor for BO.^26 27^ While existing studies on the capsule sponge test have measured people’s anxiety prior to and after undergoing the procedure and found no significant difference,^18 28 29^ these HRQL studies highlight the importance of considering the longer-term consequences of the BO diagnoses that can result from this procedure. A limitation of HRQL studies, however, may be their individualistic methodology. During my ethnographic fieldwork, I often met loved ones of people with BO who also experienced significant fear about the future possibility of cancer, highlighting the importance of considering the consequences of these diagnoses on entire communities.

Recognising that she was particularly worried, Janet’s gastroenterologist agreed to offer her more frequent surveillance endoscopies. While Janet was grateful for this provision of surveillance, it did not adequately manage her anxiety. Research often finds a lack of correlation between a person’s statistical risk of cancer and their anxiety about cancer; those objectively least at risk of cancer are not necessarily least likely to be worried about it.^30^ This means that reducing cancer risk does not necessarily manage cancer fear. It seems that fear needs to be addressed directly. The gastroenterologists’ ability to do this was limited by short appointment times. Janet had also struggled to get an appointment to see her GP, which she attributed as being due to the increase in pressure on GP surgeries in the aftermath of the COVID-19 pandemic in 2021.

Unable to see a medical professional, Janet turned to the internet to carry out her own research and discovered several support groups on Facebook for people with BO. Some of these groups were very large—consisting of more than ten thousand members—and international while others were only for UK patients. In these support groups, people shared experiences and advice regarding managing their anxiety about future cancers. They worked to reframe the condition positively, explaining that it was ‘a good thing’ to be diagnosed because surveillance could prevent cancers and cancer deaths. By writing and responding to posts on a daily basis, Janet’s peers in the support group emphasised that cancer was not an inevitable step in the trajectory of BO—it was one possible occurrence among many that could be avoided with compliance with surveillance programmes. They sought to cultivate a sense of trust in the powers of surveillance to prevent cancer—which for Janet succeeded in helping her manage her fear.

It is worth noting, however, that feeling reassured that surveillance will avert cancerous futures requires people to hold a certain level of trust in biomedical professionals. This might be more difficult for people who have experienced clinicians behaving in ways that might be deemed to be ‘untrustworthy’ for decades, such as communities who have experienced racism.^31^ For those who lack trust in healthcare practitioners, a diagnosis of cancer risk like BO can present the possibility of a cancerous future that might feel particularly inevitable.

Productive comparisons might be made between the care given to people with BO and those diagnosed with genetic cancer risk conditions. Unlike more well-known cancer risk diagnoses, such as Lynch syndrome or BRCA, BO is diagnosed on the basis of what historian Ilana Löwy calls ‘morphological risk’, meaning increased cancer risk is thought to be due to changes in the structures of tissues, rather than information gleaned from genetic testing.^32^ There are important differences between these types of risk diagnosis—inherited syndromes notably result in significantly a higher risk for patients’ family members, for example. Nevertheless, the aims of both forms of cancer risk diagnosis and the fear experienced by those diagnosed are comparable in many ways,^33^ but the care provided to people with genetics compared with morphological risk conditions differs significantly.

Genetic counselling is usually offered to people with genetic risk diagnoses, which supports them to make informed decisions about their care and to live with the uncertainty and fear brought about by the testing and any diagnoses that result. In comparison to genetic risk conditions, there has been relatively little attention given to the psychosocial consequences of morphological risk diagnoses like Barrett’s. Löwy argues that this discrepancy is the result of historical factors—genetic information was deemed to be novel in the mid-20th century and therefore triggered significant concerns regarding its consequences.^34^ In contrast, the pathological laboratory examination and endoscopy technologies used to diagnose BO have not been seen to be new and alarming in the same way, meaning that these practices have not warranted the same level of concern or the same provision of care. This paper highlights that this is perhaps an oversight on the part of researchers and clinicians.

Another factor motivating people with BO to join Facebook support groups was that they often felt that their symptoms of acid reflux were poorly managed. Some felt that these symptoms had been overlooked by biomedical professionals for whom symptoms were predominantly relevant as cancer risk factors. In contrast, for patients these sensations constituted disruptive and sometimes debilitating experiences that could significantly impair their ability to live the lives they wanted. Members of support groups shared tips to help people manage their symptoms of acid reflux, offering advice on diet, exercise and tricks such as raising the end of the bed nearest their head to alleviate heartburn at night. Because people’s anxiety about future cancers tended to be worse when symptoms flared up, learning to manage acid reflux symptoms could also help people to manage their fear of cancer.^35^ The growing popularity of Facebook support groups for people with BO, as well as other cancer risk conditions, could be seen as the product of dissatisfaction with a notion of medical care that primarily focused on reducing the risk of future disease, sometimes at the expense of addressing suffering in the present.

### Suggestions for future improvements

Even as medical practice becomes increasingly oriented towards potential futures described by statistics, current embodied experiences remain important to people who become patients. In the case of the capsule sponge test, offering information on symptom management was one way of mediating this tension between ‘patient-centred care’—concerned with the needs of the individual—and risk-centred care—primarily concerned with the statistical risk of disease. Like in other screening programmes, there remain questions about the value of screening people who are at low risk of cancer but who desire reassurance.^36^

This friction persists postdiagnosis. Updated National Institute for Health and Care Excellence guidelines published in 2023 stated that all newly diagnosed patients with BO should be offered a clinical consultation to ‘discuss risk of cancer, endoscopic surveillance plans and symptom control’ and to give the patient ‘verbal and written information about their diagnosis, available treatments and patient support groups’.^4^ The importance of addressing present as well as only potential future problems is recognised here, as is the role of support groups. However, support groups are also not currently available to all, nor are specialist nurses, charity helplines or clear resources. Currently, a division of labour occurs in which people with BO who experience high levels of worry are referred to psychological services, where they are often diagnosed with anxiety disorders. These diagnoses separate people’s fear from the medical practices that cause it, instead pathologising these patients as poorly functioning or ill. Uncertainty and fear are an inherent part of a diagnosis of cancer risk for many and must be addressed as such.

A further way to manage the fear that can be caused by screening and diagnosing BO may be to change the terminology surrounding the condition. Patient experts argue that the notion of a ‘precancer’ for many patients implies that cancer is in some sense inevitable. Instead, some advocate for BO to be described as ‘potentially precancerous’ in order to convey that cancer is one unlikely outcome among many.^37^ Historically, charities and public health campaigns have sometimes relied on ‘scare tactics’^32^ in order to motivate screening attendance. While fear can sometimes motivate healthcare engagement, some types of fear can also act as a barrier.^38^ Instead, it may be more productive to mobilise hope—focusing on the aspiration that cancer could be prevented or cured, rather than on the threat of failing to do so. This may help to address the worry that can result from efforts to diagnose risk states like BO.

## Conclusions

This paper has offered ethnographic insights into an implementation trial of the capsule sponge test. It has highlighted a tension between the aspiration to intervene in the future and the need to provide patient-centred care in the present when screening for, diagnosing and managing BO. These tensions arise when clinicians focus primarily on reducing the statistical risk of possible future disease, sometimes inadvertently overlooking patients’ needs in the present—namely, their symptoms and their fear about future cancers. Large online support groups have formed to address this gap in people’s care left by professionals, representing widespread patients’ dissatisfaction and growing distrust towards care provided by the National Health Service. As the role of risk in medicine grows further and researchers develop ever more ways to diagnose and intervene on risk, we must examine what it means empirically to provide ‘good care’ for people who are not ill—but who are ‘at risk’ of becoming ill in the future. This is particularly important as risk diagnoses transform more people into patients at a time of unprecedented pressures on healthcare services globally, raising questions about who is responsible for caring for these patients, as well as who has access to care in and beyond the clinic.

## Data Availability

No data are available.
